# Immunological Mechanisms Mediating Hantavirus Persistence in Rodent Reservoirs

**DOI:** 10.1371/journal.ppat.1000172

**Published:** 2008-11-28

**Authors:** Judith D. Easterbrook, Sabra L. Klein

**Affiliations:** The W. Harry Feinstone Department of Molecular Microbiology and Immunology, The Johns Hopkins Bloomberg School of Public Health, Baltimore, Maryland, United States of America; University of California Irvine, United States of America

## Abstract

Hantaviruses, similar to several emerging zoonotic viruses, persistently infect their natural reservoir hosts, without causing overt signs of disease. Spillover to incidental human hosts results in morbidity and mortality mediated by excessive proinflammatory and cellular immune responses. The mechanisms mediating the persistence of hantaviruses and the absence of clinical symptoms in rodent reservoirs are only starting to be uncovered. Recent studies indicate that during hantavirus infection, proinflammatory and antiviral responses are reduced and regulatory responses are elevated at sites of increased virus replication in rodents. The recent discovery of structural and non-structural proteins that suppress type I interferon responses in humans suggests that immune responses in rodent hosts could be mediated directly by the virus. Alternatively, several host factors, including sex steroids, glucocorticoids, and genetic factors, are reported to alter host susceptibility and may contribute to persistence of hantaviruses in rodents. Humans and reservoir hosts differ in infection outcomes and in immune responses to hantavirus infection; thus, understanding the mechanisms mediating viral persistence and the absence of disease in rodents may provide insight into the prevention and treatment of disease in humans. Consideration of the coevolutionary mechanisms mediating hantaviral persistence and rodent host survival is providing insight into the mechanisms by which zoonotic viruses have remained in the environment for millions of years and continue to be transmitted to humans.

Hantaviruses are negative sense, enveloped RNA viruses (family: Bunyaviridae) that are comprised of three RNA segments, designated small (S), medium (M), and large (L), which encode the viral nucleocapsid (N), envelope glycoproteins (G_N_ and G_C_), and an RNA polymerase (Pol), respectively. More than 50 hantaviruses have been found worldwide [1]. Each hantavirus appears to have coevolved with a specific rodent or insectivore host as similar phylogenetic trees are produced from virus and host mitochondrial gene sequences [2]. Spillover to humans causes hemorrhagic fever with renal syndrome (HFRS) or hantavirus cardiopulmonary syndrome (HCPS), depending on the virus [3]–[5]. Although symptoms vary, a common feature of both HFRS and HCPS is increased permeability of the vasculature and mononuclear infiltration [4]. Pathogenesis of HRFS and HCPS in humans is hypothesized to be mediated by excessive proinflammatory and CD8+ T cell responses (Table 1 [Table ppat-1000172-t002]).

**Table 1:** Summary of Immune Responses in Humans during Hantavirus Infection.

Categorical Response	Immune Marker	Effect of Infection	Virus Species^a^	In Vitro/In Vivo	Tissue or Cell Type^b^, Phase of Infection^c^	References
**Innate**	RIG-I	Elevated	SNV	In vitro	HUVEC, ≤24 h p.i.	[79]
		Reduced	NY-1V	In vitro	HUVEC, ≤24 h p.i.	[37]
	TLR3	Elevated	SNV	In vitro	HUVEC, ≤24 h p.i.	[79]
	IFN-β	Elevated	PUUV, PHV, ANDV	In vitro	HSVEC, HMVEC-L, ≤24 h p.i.	[36],[80]
		Reduced	TULV, PUUV NSs	In vitro	COS-7 and MRC5 cells, ≤24 h p.i.	[32],[33]
	IFN-α	Elevated	PUUV, HTNV	In vitro	MФ, DCs, 4 days p.i.	[30]
		No change	HTNV	In vivo	Blood, acute	[81]
	IRF-3, IRF-7	Elevated	SNV, HTNV, PHV, ANDV	In vitro	HMVEC-L, ≤24 h p.i.	[33],[38]
	MxA	Elevated	HTNV, NY-1V, PHV, PUUV, ANDV, SNV, TULV	In vitro	MФ,HUVEC,HMVEC-L, 6 h–4 days p.i.	[36], [39]–[41],[79]
	MHC I and II	Elevated	HTNV	In vitro	DCs, 4 days p.i.	[30]
	CD11b	Elevated	PUUV	In vivo	Blood, acute	[82]
	CD40, CD80, CD86	Elevated	HTNV	In vitro	DCs, 4 days p.i.	[30],[83]
	NK cells	Elevated	PUUV	In vivo	BAL, acute	[84]
**Proinflammatory/Adhesion**	IL-1β	Elevated	SNV, HTNV	In vivo	Blood, lungs, acute	[85],[86]
	IL-6	Elevated	SNV, PUUV	In vivo	Blood, lungs, acute	[85],[87],[88]
	TNF-α	Elevated	PUUV, SNV, HTNV	In vivo	Blood, lungs, kidney, acute	[85],[86],[88],[89]
		Elevated	HTNV	In vitro	DCs, 4 days p.i.	[30]
	CCL5	Elevated	SNV, HTNV	In vitro	HMVEC-L, HUVEC, 12 h–4 days p.i.	[38],[39],[90]
	CXCL8	Elevated	PUUV	In vivo	Blood, acute	[82]
		Elevated	PUUV	In vivo	Men, blood, acute	[62]
		Elevated	TULV, PHV, HTNV	In vitro	HUVEC, MФ, 2–4 days p.i.	[39],[91]
	CXCL10	Elevated	SNV, HTNV, PHV	In vitro	HMVEC-L,HUVEC, 3–4 days p.i.	[38],[39]
		Elevated	PUUV	In vivo	Men, blood, acute	[62]
	IL-2	Elevated	SNV, HTNV, PUUV	In vivo	Blood, lungs, acute	[82],[86]
	Nitric oxide	Elevated	PUUV	In vivo	Blood, acute	[92]
	GM-CSF	Elevated	PUUV	In vivo	Women, blood, acute	[62]
	ICAM, VCAM	Elevated	PUUV	In vivo	Kidney, acute	[87]
		Elevated	HTNV, PHV	In vitro	HUVEC, 3–4 days p.i.	[30],[39]
	E-selectin	Elevated	PUUV	In vivo	Blood, acute	[82]
**CD8+ and CD4+ T cells**	IFN-γ	Elevated	HTNV, SNV	In vivo	Blood, CD4+,CD8+, lungs, acute	[81],[86]
	CD8+	Elevated	DOBV, PUUV, HTNV	In vivo	Blood, BAL, acute	[52],[84],[93]
	Virus-specific IFN-γ+CD8+	Elevated	PUUV, SNV	In vivo	PBMC, acute	[45],[94]
	Perforin, Granzyme B	Elevated	PUUV	In vivo	Blood, acute	[95]
	CD4+CD25+ “activated”	Elevated	DOBV, PUUV	In vivo	PBMC, acute	[89],[93]
	IL-4	Elevated	SNV	In vivo	Lungs, acute	[86]
**Regulatory**	“suppressor T cells”^d^	Reduced	HTNV	In vivo	Blood, acute	[52]
	IL-10	Elevated	PUUV	In vivo	Blood, acute	[86]
	TGF-β	Elevated	PUUV	In vivo	Kidney, acute	[89]
**Humoral**	IgM, IgG, IgA, IgE	Elevated	All hantaviruses	In vivo	Blood	[4]

a SNV, Sin Nombre virus; NY-1V, New York-1 virus; PUUV, Puumala virus; PHV, Prospect Hill virus; ANDV, Andes virus; TULV, Tula virus; HTNV, Hantaan virus; DOBV, Dobrava virus.

b HUVEC, human umbilical vascular endothelial cells; HSVEC, human saphenous vein endothelial cells; HMVEC-L, human lung microvascular endothelial cells; COS-7, African green monkey kidney fibroblasts transformed with Simian virus 40; MRC5, human fetal lung fibroblasts; MФ, macrophages; DCs, dendritic cells; BAL, bronchoalveolar lavage, PBMC, human peripheral blood mononuclear cells.

c Acute infection is during symptomatic disease in patients.

d Suppressor T cells likely represent cells currently referred to as regulatory T cells.

**Table 2 ppat-1000172-t002:** Summary of Immune Responses in Rodents during Hantavirus Infection.

Categorical Response	Immune Marker	Effect of Infection	Virus Species^a^	Host, Tissue or Cell Type^b^	Phase of Infection^c^	References
**Innate**	TLR7	Reduced	SEOV	Male Norway rats, lungs	Acute, Persistent	[19]
		Elevated	SEOV	Female Norway rats, lungs	Acute, Persistent	[19]
	RIG-I	Elevated	SEOV	Female Norway rats, lungs	Acute, Persistent	[19]
		Elevated	SEOV	Newborn rats, thalamus	Acute	[96]
	TLR3	Elevated	SEOV	Male Norway rats, lungs	Acute, Persistent	[19]
	IFN-β	Reduced	SEOV	Male Norway rats, lungs	Acute, Persistent	[19],[61]
		Elevated	SEOV	Female Norway rat lungs	Acute	[19],[61]
	Mx2	Reduced	SEOV	Male Norway rats, lungs	Acute, Persistent	[19],[60]
		Elevated	SEOV	Female Norway rats, lungs	Acute, Persistent	[19],[60]
		Elevated	HTNV, SEOV	Mice^d^, fibroblasts transfected with Mx2	3–4 days p.i.	[97]
	JAK2	Elevated	SEOV	Female Norway rats, lungs	Acute	[60]
	MHC II	Elevated	PUUV	Bank voles	Genetic susceptibility	[74]
**Proinflammatory/Adhesion**	IL-1β	Reduced	SEOV	Male Norway rats, lungs	Persistent	[29]
	IL-6	Reduced	SEOV	Male and female Norway rats, lungs	Acute, Persistent	[29],[61]
		Elevated	SEOV	Male rats, spleen	Acute	[29]
	TNF-α	Reduced	HTNV	Newborn mice^d^, CD8+, spleen	Acute	[49],[50]
		Reduced	SEOV	Male Norway rats, lungs	Acute, Persistent	[29],[42],[61]
		Elevated	SEOV	Female Norway rats, lungs	Persistent	[61]
	CX3CL1, CXCL10	Reduced	SEOV	Male Norway rats, lungs	Acute, Persistent	[29]
		Elevated	SEOV	Male Norway rats, spleen	Acute	[29]
	CCL2, CCL5	Elevated	SEOV	Male Norway rats, spleen	Acute	[29]
	NOS2	Reduced	SEOV	Male Norway rats, lungs	Acute, Persistent	[29],[61]
		Elevated	SEOV	Male Norway rats, spleen	Acute	[29]
		Elevated	HTNV	Mouse MФ^d^, in vitro	6 h p.i.	[98]
	VCAM, VEGF	Elevated	SEOV	Male Norway rats, spleen	Acute	[29]
**CD8+ and CD4+ T cells**	CD8+	Reduced	HTNV	Newborn mice^d^, spleen	Persistent	[50]
		Elevated	HTNV	SCID mice^d^, CD8+ transferred, spleen	Persistence	[49]
		Elevated	SEOV	Female Norway rats, lungs	Persistent	[61]
	IFN-γ	Elevated	SEOV	Female Norway rats, lungs	Persistent	[61]
		Elevated	SEOV	Male Norway rats, spleen	Acute	[29]
		Elevated	SEOV	Male and female Norway rats, splenocytes	Acute	[20]
		Elevated	SNV	Deer mice, CD4+ T cells	Acute	[48]
		Elevated	HTNV	Newborn mice^d^, CD8+ T cells, spleen	Acute	[50]
		Reduced	HTNV	Newborn mice^d^, CD8+ T cells, spleen	Persistent	[99]
	IFN-γR	Elevated	SEOV	Female Norway rats, lungs	Acute, Persistent	[60]
		Reduced	SEOV	Male Norway rats, lungs	Persistent	[60]
	T cells	Elevated	SEOV	Nude rats	Persistence	[47]
		Elevated	HTNV	Nude mice^d^	Persistence	[100]
	IL-4	Reduced	SEOV	Male Norway rats, lungs	Acute, Persistent	[61]
		Elevated	SNV	Deer mice, CD4+ T cells	Acute	[48]
		Elevated	SEOV	Male and female Norway rats, splenocytes	Acute	[20]
**Regulatory**	Regulatory T cells	Elevated	SEOV	Male Norway rats, lungs	Persistent	[42],[61]
	FoxP3	Elevated	SEOV	Male Norway rats, lungs	Persistent	[29],[42],[61]
	TGF-β	Elevated	SEOV	Male Norway rats, lungs	Persistent	[29]
			SNV	Deer mice, CD4+ T cells	Persistent	[48]
	IL-10	Reduced	SEOV	Male Norway rats, lungs and spleen	Acute, Persistent	[29]
		Elevated	SNV	Deer mice, CD4+ T cells	Acute	[48]
**Humoral**	IgG	Elevated	SNV	Deer mice	Persistent	[12],[57]
		Elevated	SEOV	Norway rats	Persistent	[16],[17]
		Elevated	HTNV	Field mice	Persistent	[15]
		Elevated	PUUV	Bank voles	Persistent	[14]
		Elevated	BCCV	Cotton rats	Persistent	[18],[58]

a SEOV, Seoul virus; HTNV, Hantaan virus, PUUV, Puumala virus; SNV, Sin Nombre virus; PUUV, Puumala virus; BCCV, Black Creek Canal virus.

b MФ, macrophages.

c Acute infection is <30 days p.i. and persistent infection is ≥30 days p.i.

d *Mus musculus*, non-natural reservoir host for hantaviruses.

In contrast to humans, hantaviruses persistently infect their reservoir hosts, presumably causing lifelong infections [6]. Hantaviruses are shed in saliva, urine, and feces, and transmission among rodents or from rodents to humans occurs by inhalation of aerosolized virus in excrement or by transmission of virus in saliva during wounding [7],[8]. Although widely disseminated throughout the rodent host, high amounts of hantaviral RNA and antigen are consistently identified in the lungs of their rodent hosts, suggesting that the lungs may be an important site for maintenance of hantaviruses during persistent infection [9]–[18]. Hantavirus infection in rodents is characterized by an acute phase of peak viremia, viral shedding, and virus replication in target tissues, followed by a persistent phase of reduced, cyclical virus replication despite the presence of high antibody titers (Figure 1) [12]–[16], [18]–[20]. The onset of persistent infection varies across hantavirus–rodent systems, but generally the acute phase occurs during the first 2–3 weeks of infection and virus persistence is established thereafter (Figure 1).

**Figure 1:**
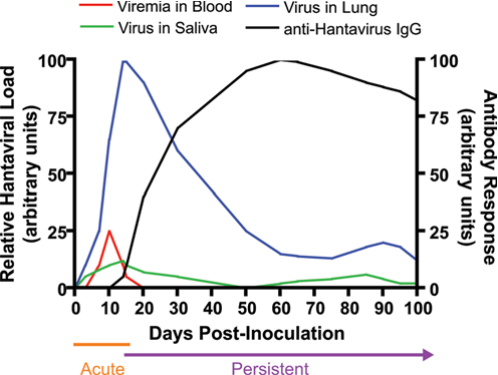
Kinetics of Hantavirus Infection in Rodents. Adapted from Lee et al. [15] and others [12]–[14],[16],[18],[20], the kinetics of relative hantaviral load in blood (red), saliva (green), and lung tissue (blue) and antibody responses (black) during the acute and persistent phases of infection are represented. The amount of genomic viral RNA, infectious virus titer, and/or relative amount of viral antigen have been incorporated as relative hantaviral load. The antibody response is integrated as the relative amount of anti-hantavirus IgG and/or neutralizing antibody titers.

Hantavirus infection alone does not cause disease, as reservoir hosts and non-natural hosts (e.g., hamsters infected with Sin Nombre virus [SNV] or Choclo virus) may support replicating virus in the absence of overt disease [12],[14],[16],[18],[21],[22]. Our primary hypothesis is that certain immune responses that are mounted in humans during hantavirus infection are suppressed in rodent reservoirs to establish and maintain viral persistence, while preventing disease (Table 2). During the coevolution of hantaviruses with their reservoir hosts, the viruses may have evolved mechanisms to enhance persistence, including immune evasion, direct suppression or alteration of host antiviral, proinflammatory, and cellular immune responses, and induction of host regulatory responses. Alternatively, hosts may have evolved adaptations, including immunological responsiveness to steroid hormones and pre-existing host genetic factors, to regulate the detrimental effects of infection, which also may affect persistence of hantaviruses.

## Virus-Mediated Responses to Hantaviruses

### 

#### Immune evasion

Mechanisms of immune evasion, including viral mutation and segment reassortment, may contribute to viral persistence. Cyclical rises of Seoul virus (SEOV) L segment deletions and S segment insertions in regions necessary for initiation of transcription are observed in vitro [6]; whether mutated RNAs can self-repair and whether this occurs in vivo remains to be determined. Quasispecies have been identified in vivo in deer mice (*Peromyscus maniculatus*) and European common voles (*Microtus arvalis*) infected with SNV and Puumala virus (PUUV), respectively, with nucleotide and amino acid mutations identified in immunodominant regions of the G_N_ protein [23],[24]. Segment reassortment of hantaviruses has been identified in Vero E6 cells that are co-infected with related strains of SNV and in vivo in deer mice infected with SNV [25],[26]. Recent data also suggest that a newly discovered hantavirus in Paraguay may be a reassortment between Laguna Negra or Rio Mamoré viruses with Pergamino or Maciel viruses [27]. Quasispecies and reassortants likely contribute to the evolution of new species of hantaviruses, but also may be involved in immune evasion.

#### Direct alteration of host cellular responses

Hantaviruses can infect macrophages and endothelial cells in rodents and humans [13],[14],[16],[28]. These cell types have been identified primarily by morphological analyses or by non-specific cell separation. Recently, SEOV N protein was definitively identified in macrophages and endothelial cells in lung tissue of infected rats using cell-specific antibodies [29]. Hantaan virus (HTNV) infects human dendritic cells (DCs) in vitro and induces DC activation and maturation [30]. Whether rodent DCs are infected by hantaviruses and whether rodent macrophage and DC activity is suppressed by hantavirus infection to cause persistence remains unknown. Several viruses in the Bunyaviridae family encode a non-structural protein (NSs) that suppresses antiviral immune responses in infected cells [31]. The S segment of PUUV and Tula virus (TULV) has an open reading frame (ORF) for NSs, a protein that reduces the expression of IFN-β in human lung fibroblasts and contributes to virus survival in vitro [32],[33]. Immunoreceptor tyrosine-based activation motifs (ITAMs) that bind kinases to regulate immune and endothelial cell function have been identified in the G_N_ protein of hantaviruses [34],[35]. Additionally, the cytoplasmic tails of HCPS-causing hantavirus G proteins inhibit the expression of type I IFN responses in human umbilical vein endothelial cells (HUVECs) and human lung microvascular endothelial cells (HMVEC-Ls) in vitro [36],[37]. Whether hantavirus proteins and RNA have immunomodulatory activity in rodent reservoirs remains unknown.

## Host-Mediated Responses to Hantaviruses

### 

#### Innate antiviral and proinflammatory responses

Infection of HMVEC-Ls and HUVECs indicate that at least some of the hantaviruses that cause disease in humans (e.g., HTNV, Andes virus [ANDV], SNV, New York-1 virus [NY-1V], PUUV, and SEOV) delay induction of factors in the type I IFN pathway (e.g., production of IFN-α, IFN-β, and MxA) as compared with hantaviruses that cause no known disease in humans (e.g., TULV and Prospect Hill virus [PHV]) [36], [38]–[41]. Delayed production of antiviral responses in humans may contribute to more efficient replication of pathogenic hantaviruses than non-pathogenic hantaviruses. Despite delayed induction of type I IFNs, human cells mount innate antiviral responses that also may contribute to viral clearance.

In the lungs of rats with high amounts of virus (i.e., male rats), the expression of pattern recognition receptors (i.e., *Rig-I* and *Tlr7*) is reduced or remains unchanged throughout SEOV infection, suggesting that inhibition of viral recognition may contribute to the establishment of persistent infection [19]. Furthermore, antiviral (e.g., IFN-β, Mx2, and IFN-γ) and proinflammatory (e.g., IL-1β, TNF-α, and NOS2) responses are reduced or unaltered during infection in the lungs of male rats, which also may contribute to hantavirus persistence [19],[29],[42]. In contrast, in the spleen, a peripheral immune organ that supports low amounts of virus, the expression and production of proinflammatory and antiviral factors are elevated during acute SEOV infection and subsequently return to baseline [20],[29]. Thus, rats infected with SEOV do not appear to be globally immunosuppressed, but rather have a site-specific reduction of proinflammatory responses. There is no evidence that male rats that are naturally infected with SEOV are more likely to acquire additional pathogens, further illustrating that infected rats are not immunocompromised [43]. Conversely, natural populations of deer mice that have antibody against SNV elicit a lower response to phytohemagglutinin (i.e., a measure of immunocompetence) than their uninfected counterparts, suggesting that SNV causes some degree of immunosuppression in deer mice [44]. Administration of exogenous IL-1β, which elevates circulating IL-1β and *Il6* and *Tnfα* expression in the lungs within physiological ranges, does not affect SEOV persistence in male rats nor does it cause observable disease [29]. Thus, extremely high proinflammatory responses observed during acute infection in humans may be necessary for viral clearance at the expense of causing potentially fatal proinflammatory-mediated disease.

#### CD4+ and CD8+ T cell responses

Cellular immune responses, in particular CD8+ T cells, contribute to clearance of hantaviruses in humans at the expense of causing disease [45],[46]. Following inoculation with SEOV, nude (i.e., T cell deficient) rats have more virus in target tissues and shed more infectious virus than do their immunocompetent counterparts and die 10 weeks after inoculation, indicating that T cells contribute to the control of virus replication and survival in a reservoir host [47]. During the acute phase of SNV infection, deer mice have observable Th1 and Th2 responses (i.e., elevated expression of *Ifnγ*, *Gata3* [i.e., the hallmark Th2 transcription factor], *Il4*, and *Il5*) in cultured CD4+ T cells, which are not evident in CD4+ T cells isolated from persistently infected deer mice [48]. In severe-combined immunodeficient (SCID) mice (*Mus musculus*), the transfer of functional CD8+ T cells is necessary for clearance of HTNV [49]. Persistence of HTNV in newborn BALB/c mice is correlated with a decrease in HTNV-specific CD8+ T cell numbers and activity, as measured by IFN-γ production, further suggesting a role of functional CD8+ T cell responses in viral clearance [49],[50]. Not only are laboratory mice non-natural hosts, but SCID and newborn mice do not have fully functional immune systems, so these models do not accurately represent viral persistence in immunocompetent rodent reservoir populations. The effect of infection on hantavirus-specific CD8+ T cells in adult rodent reservoir hosts requires examination.

#### Regulatory T cell responses

Regulatory T cell responses suppress proinflammatory and effector T cell responses locally at the site of infection to allow pathogen persistence, as well as to mitigate proinflammatory-mediated pathogenesis [51]. Recent studies have demonstrated that regulatory T cells contribute to SEOV and SNV persistence in rats and deer mice, respectively [42],[48]. Expression of *Foxp3* mRNA and proportions of CD4+CD25+FoxP3+ regulatory T cells are elevated locally at a site of elevated SEOV replication (i.e., in the lungs) in male rats during persistent SEOV infection [29],[42]. Functional inactivation of regulatory T cells reduces the amount of SEOV RNA present in the lungs and the proportion of animals shedding viral RNA in saliva [42]. In the lungs, the expression and production of TGF-β is elevated and TNF-α is suppressed during persistent infection; both cytokine expression patterns are dependent on the presence of functional regulatory T cells [42]. Similarly, CD4+ T cells isolated from deer mice during the persistent phase of SNV infection have higher expression of *Tgfβ* than do CD4+ T cells isolated from deer mice during the acute phase of infection [48]. The production of IL-10 is consistently reduced throughout SEOV and during the persistent phase of SNV infection in rats and deer mice, respectively, revealing that IL-10 does not contribute to regulatory T cell–mediated hantaviral persistence [29],[42],[48]. Because responses to hantavirus infection in humans involve overproduction of proinflammatory cytokines, it is consistent that “regulatory T cell activity” (i.e., T cells which reduce ConA-induced proliferation of PBMCs) is suppressed during symptomatic HTNV infection in humans [52]; whether suppressed regulatory T cell responses contribute to disease in humans requires consideration.

#### Antibody responses

Hantaviruses persist in their rodent hosts despite the presence of neutralizing antibody. Antibody against hantaviruses is usually detectable after the first 2 weeks of infection, rapidly increases for the next 4–6 weeks, and declines, but remains detectable presumably for the lifetime of the rodent (Figure 1) [9], [13]–[16],[18],[29]. Hantavirus-specific antibody responses, although not capable of eliminating virus, can serve a protective role against infection. Maternal antibody protects offspring of hantavirus-infected dams during the first 2 months of life (i.e., when the immune system is not fully developed) in various rodent reservoirs [53]–[55]. Not only are young rodents protected from hantavirus infection, but young male and female bank voles with maternal antibody against PUUV also mature earlier, suggesting that reproductive success may be increased in bank voles with, as compared to bank voles without, maternal antibody [54]. How hantaviruses evade antibody responses in their rodent hosts remains to be answered.

#### Sex differences and sex steroids

In natural populations of rodent reservoirs, males are more frequently infected with hantaviruses and are more likely to engage in aggressive encounters than are females, which may result in elevated exposure and transmission of hantaviruses among males [10], [56]–[59]. In laboratory settings, when given the same challenge, male rats have more SEOV RNA and antigen in target organs and saliva than do females [19],[20]. Removal of the testes in males (i.e., reduction of androgens to non-detectable levels) reduces, whereas removal of the ovaries in females (i.e., reduction of estrogens and progesterone to non-detectable levels) increases SEOV RNA loads as compared with their intact counterparts [19]. Consistent with sex differences in SEOV load, the expression of innate antiviral (e.g., *Tlr7*, *Myd88*, *Rig-I*, *Visa*, *Ifnβ*, and *Mx2*) and proinflammatory (e.g., *Tnfα* and *Ccl5*) factors is higher in the lungs of female than male rats [11],[19],[60],[61]. Similarly, immunocompetence, as measured by swelling in response to PHA, is higher in female than in male deer mice during SNV infection [44]. Conversely, the expression and production of regulatory factors, including *Foxp3* and TGF-β, is elevated in the lungs of males as compared with those of females [61]. These sexually dimorphic immune responses may be dependent on estradiol in females and testosterone in males, as gonadectomy reverses these differences [19],[60]. It is plausible that reduced innate and proinflammatory defenses and elevated regulatory responses combined with an increased propensity to engage in aggression may contribute to increased maintenance and transmission of hantaviruses among male as opposed to among female rodents. Whether there exists a sexual dimorphism in the risk of zoonotic transmission of hantaviruses should continue to be considered.

Sex differences in response to hantavirus infection in humans are beginning to receive attention. During acute PUUV infection in humans, circulating concentrations of CXCL8 and CXCL10 are higher, whereas concentrations of IL-9 and GM-CSF are lower in men than in women [62]. Although a similar proportion of men and women have antibodies against PUUV [63], men are more likely to develop symptoms of disease (i.e., be hospitalized) during PUUV infection than are women [62],[64]. Whether sexually dimorphic immune responses during hantavirus infection cause differences in the severity of disease between men and women requires further investigation.

#### Glucocorticoids

Glucocorticoids are potent immunosuppressive steroid hormones that can suppress proinflammatory and cellular responses and have been administered to patients with HFRS or HCPS to reduce immunopathology [65],[66]. Infection of humans with either HTNV or PUUV causes pituitary and adrenal necrosis, which may contribute to the reduced concentrations of cortisol and elevated proinflammatory responses in patients during the acute phase of infection [67],[68]. In rats, circulating concentrations of corticosterone are reduced during SEOV infection in males, but not in females [61]. Males with reduced concentrations of glucocorticoids have more SEOV RNA in the lungs than do males with elevated concentrations [61]. No such relationship between glucocorticoid concentration and SEOV load is observed in females [61]. Low concentrations of corticosterone correlate with elevated regulatory responses (i.e., expression of *Foxp3* and production of TGF-β) and expression of the glycogenase, matrix metalloprotease (*Mmp*)-*9*, in the lungs of male, but not female, rats during SEOV infection [61]. Increased production of MMP-9 may disrupt the basement membrane and extracellular matrices in tissues to increase virus dissemination in male rats [69]. Whether hantaviruses alter glucocorticoids in other reservoir hosts requires further investigation. Based on our data, administration of corticosteroids to patients with HFRS or HCPS would not be expected to cause chronic hantavirus infection and may even reduce viral dissemination in tissues.

#### Genetic factors

Host genetic factors may contribute to susceptibility to and outcome of hantavirus infection in both humans and rodent hosts. Patients with TNF polymorphisms associated with elevated or reduced TNF-α transcription are predisposed to exhibit more severe disease during PUUV infection [70],[71]. Possession of certain HLA haplotypes (i.e., HLA B8-DR3, C4A*Q0, and DRB1*0301) is a risk factor for severe disease during PUUV infection in humans, illustrating that antigen presentation and T cell responses likely contribute to the severity of disease [72],[73]. Several MHC alleles may alter susceptibility in rodent reservoirs, specifically Cgl-*DQA*-09, which is positively, and Cgl-*DQA*-05 and Cgl-*DQA*-12, which are negatively, associated with the likelihood of bank voles being infected with PUUV [74]. Because uninfected bank voles may never have been exposed to PUUV, whether these specific MHC alleles alter susceptibility following a known exposure to PUUV remains to be determined.

## Conclusions

Hantaviruses and their rodent reservoirs represent highly coevolved systems in which virulence and host responses have been adapted to ensure survival of both the virus and the host. The current literature suggests that hantavirus persistence is mediated by both the virus and the host. Although several potential mechanisms mediating the persistence of hantaviruses in their rodent hosts have been discovered in recent years, there are a number of intriguing questions that remain to be addressed:

- Do hantaviral NSs inhibit type I IFN responses in rodents and therefore contribute to viral persistence?
- Is DC and macrophage activity (e.g., antigen presentation, cytokine production, and T cell activation) suppressed by hantavirus infection in rodent reservoirs?
- Does suppression of excessive proinflammatory cytokine responses (e.g., TNF-α) prevent disease in rodents at the cost of causing viral persistence?
- Is the activity of hantavirus-specific CD8+ T cells suppressed during infection to mediate viral persistence in rodents?
- How is regulatory T cell activity induced by hantaviruses in reservoir hosts?
- What is the mechanism of regulatory T cell–mediated hantaviral persistence (e.g., suppression of proinflammatory and/or CD8+ T cell activity)?
- Do host genetic factors, in addition to MHC alleles, contribute to the susceptibility of rodents to hantaviruses?
- What is the role of non-immune mediators, including MMP-9 [61] and receptor use for cellular entry [75],[76], in hantaviral dissemination and persistence in rodents?

We propose that comparing immune responses in rodents to those in humans may provide insight into ways to prevent pathology in humans. Although advances are being made in the development of a hantavirus vaccine, there currently is no FDA-approved vaccine or drug for prevention or treatment of hantaviral disease [77],[78]. Elevated regulatory T cell responses in rodents contribute to hantavirus persistence, possibly by suppressing proinflammatory responses (i.e., production of TNF-α) [42],[48]. Regulatory T cell responses during hantavirus infection have not been well characterized in humans, but may be downregulated [52] and contribute to symptoms of HFRS and HCPS. Targeted manipulation of regulatory T cell responses by adoptive transfer of regulatory T cells, administration of anti-TNFα therapy, or treatment with glucocorticoids may control the “cytokine storm” that is initiated when hantaviruses infect humans and cause severe immunopathology. Understanding the mechanisms mediating viral persistence in the absence of disease in reservoir hosts may contribute to advances in the treatment of HFRS and HCPS in humans.
